# PKU mutation p.G46S prevents the stereospecific binding of l‐phenylalanine to the dimer of human phenylalanine hydroxylase regulatory domain

**DOI:** 10.1002/2211-5463.12175

**Published:** 2017-01-21

**Authors:** João Leandro, Jaakko Saraste, Paula Leandro, Torgeir Flatmark

**Affiliations:** ^1^ Department of BiomedicineUniversity of BergenNorway; ^2^ Metabolism and Genetics GroupResearch Institute for Medicines and Pharmaceutical Sciences (iMed.UL)Faculty of PharmacyUniversity of LisbonPortugal

**Keywords:** βαββαβ folds, phenylalanine hydroxylase, regulatory domain

## Abstract

Mammalian phenylalanine hydroxylase (PAH) has a potential allosteric regulatory binding site for l‐phenylalanine (l‐Phe), in addition to its catalytic site. This arrangement is supported by a crystal structure of a homodimeric truncated form of the regulatory domain of human PAH (hPAH‐RD ^1–118/19–118^) [Patel D *et al*. (2016) *Sci Rep* doi: 10.1038/srep23748]. In this study, a fusion protein of the domain (MBP‐(pep_Xa_)‐hPAH‐RD ^1–120^) was overexpressed and recovered in a metastable and soluble state, which allowed the isolation of a dimeric and a monomeric fusion protein. When cleaved from MBP, hPAH‐RD forms aggregates which are stereospecifically inhibited by l‐Phe (> 95%) at low physiological concentrations. Aggregation of the cleaved dimer of the mutant form hPAH‐G46S‐RD was not inhibited by l‐Phe, which is compatible with structurally/conformationally changed βαββαβ ACT domain folds in the mutant.

**Abbreviations**
ANS: 8‐anilino‐1‐naphthalenesulfonic acid
MBP: maltose‐binding protein
*n*_H_: Hill coefficient
PAH: phenylalanine hydroxylase
RD: regulatory domain
SAXS: small angle X‐ray scattering
SEC: size‐exclusion chromatography

Enzyme kinetic and biophysical studies of the full‐length rat and human phenylalanine hydroxylase (r/hPAH) homotetramer have indicated that its catalytic activation by l‐phenylalanine (l‐Phe) involves a slow (*s*‐to‐*min* timescale) global conformational change, preceding the chemical steps characteristic of a hysteretic enzyme 1, 2. Mammalian PAH shows a complex activation mechanism. Based on indirect experimental evidence, two main working models have been proposed: (i) binding of l‐Phe to a putative allosteric site in the N‐terminal regulatory domain (RD) as well as to the catalytic site 3, 4, 5, 6, 7, 8, and (ii) cooperative binding of l‐Phe (*n*_H_ about 2) at the catalytic site which represents the site of initiation (‘epicentre’) for the conformational transition in the activation process 9, 10, 11, 12, 13, 14. The first model was originally based on indirect enzyme kinetic and biophysical studies on the rPAH tetramer and truncated RD constructs, but has lately gained further support from the determination of the X‐ray crystal structure of the full‐length ligand‐free and autoinhibited rat and human enzyme at low resolution (PDB ID: 5DEN at 2.9 Å 15 and PDB ID: 5EGQ at 3.6 Å) 8) and the high resolution crystal structure (PDB ID: 5FII at 1.8 Å) of a homodimeric truncated form of the human RD (hPAH‐RD) 16. Representing the key finding of this study, the structure revealed two l‐Phe molecules bound to a homodimer at the interphase of the two β_1_α_1_β_2_β_3_α_2_β_4_ ACT domain folds along the plane of the twofold axis 16. However, in the absence of l‐Phe, the overexpressed construct aggregated.

The missense mutation p.G46S in hPAH (Fig. 1) is associated with a severe form of phenylketonuria and generates a misfolded protein which is rapidly degraded on expression in HEK293 cells 17. The crystal structure of the wt‐hPAH‐RD has shown that l‐Phe binding includes the sequence region E^43^xVxAL in the two protomers 16, and it was therefore of great interest to test the effect of a G→S substitution on the l‐Phe binding to the β_1_α_1_β_2_β_3_α_2_β_4_ ACT domain folds in the hPAH‐G46S‐RD mutant form. Since a previous attempt has failed to express this mutant form in a nonaggregated and soluble form 16, we here overexpressed it as a fusion protein (MBP‐(pep)_Xa_‐hPAH‐RD). This change in the RD construct results in a stabilization of both the wt and mutant forms of the RD protein in a metastable and soluble state, and allows the isolation of a dimeric and a monomeric fusion protein. When cleaved by factor Xa, the maltose‐binding protein (MBP)‐free wt‐RD of both forms undergoes aggregation, which is stereospecifically prevented by l‐Phe. Thus, the substrate stabilizes a dimer of the RD. 8‐Anilino‐1‐naphthalenesulfonic acid (ANS) binding studies with the dimeric wt fusion protein confirmed the stereospecific binding of l‐Phe in a physiological concentration range. With reference to the behaviour of the wt‐hPAH‐RD, it is shown that l‐Phe does not bind and stabilize the mutant form hPAH‐G46S‐RD due to a structural/conformational change in its wt‐binding site involving residues E^43^xVxAL in the dimeric β_1_α_1_β_2_β_3_α_2_β_4_ ACT domain folds.

**Figure 1:**
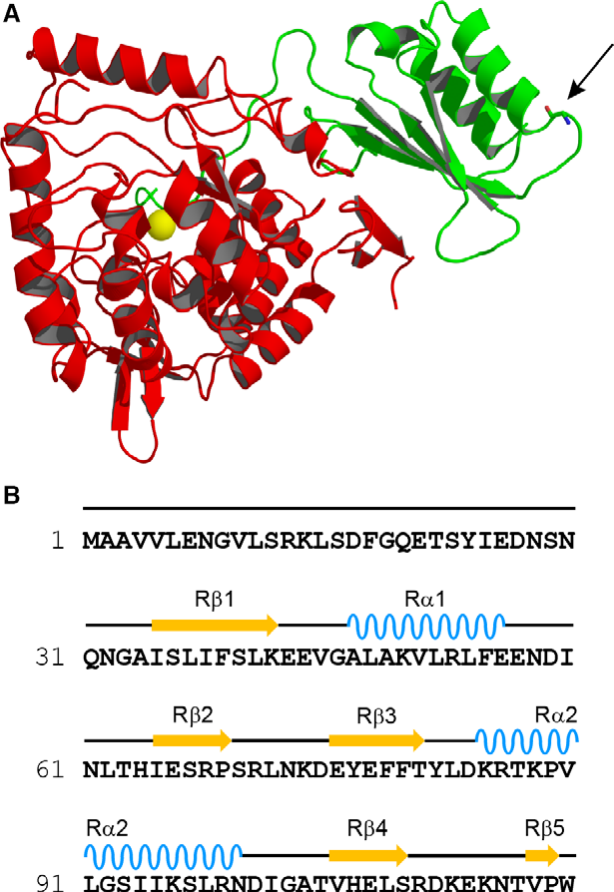
3D structure of PAH protomer (rPAH ^1–429^), sequence and secondary structure assignment of the N‐terminal regulatory domain (RD) of hPAH and the localization/interactions of the G46 residue in the RD. (A) Ribbon representation of the regulatory/catalytic domain crystal structure of rPAH (PDB ID: 1PHZ at the highest resolution (2.2 Å) for this structure) in the monomeric form with the RD shown in green, the catalytic domain in red, the iron as a yellow sphere and G46 in stick model (pointed arrow). (B) Sequence of the N‐terminal regulatory domain of hPAH (SwissProt P00439) with elements of secondary structure (determined from the coordinates of PDB ID: 1PHZ with the program DSSP 26 indicated above the sequence and numbered sequentially from the N terminus 4. Figure (A) was created using PyMOL, version 1.1 (DeLano Scientific) 27.

## Materials and methods

TB1 cells, the prokaryotic expression vector pMAL‐c2/pMAL‐hPAH and the amylose resin were obtained from New England Biolabs (Ipswich, MA, USA). The restriction protease factor Xa was obtained from Protein Engineering Technology ApS (Aarhus, Denmark). ANS were obtained from Sigma‐Aldrich (Oslo, Norway).

### Site‐specific mutagenesis

The wt‐RD (pMAL‐hPAH‐RD^1–120^) and its G46S mutant form (pMAL‐hPAH‐G46S‐RD^1–120^) were obtained by introducing a stop signal in codon 121 of hPAH by site‐directed mutagenesis (QuikChange^®^ II; Stratagene, Santa Clara, CA, USA), using the wt‐pMAL‐hPAH 18 and pMAL‐G46S‐hPAH constructs 17 as templates respectively. Primers 5ʹ‐GACACAGTGCCCTGGT**AA**CCAAGAACCATTCAAGAGC‐3ʹ (forward) and 5ʹ‐GCTCTTGAATGGTTCTTGG**TT**ACCAGGGCACTGTGTC‐3ʹ (reverse) used for mutagenesis were provided by Eurogentec (Seraing, Belgium; the mismatch nucleotides are shown in bold). The authenticity of the mutagenesis was verified by DNA sequencing as described previously 17.

### Overexpression and isolation of fusion proteins

**Figure 2:**
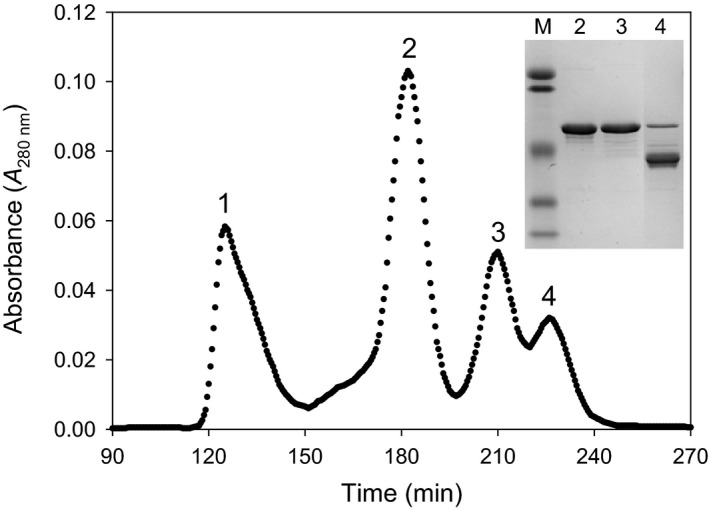
Size‐exclusion chromatography of the MBP‐hPAH‐RD ^1–120^ construct. Peak 1, higher order oligomeric forms (eluted at the void volume); peak 2, dimeric form (~ 156 kDa); peak 3, monomeric form (~ 65 kDa), and peak 4, degradation products (~ 39 kDa). The molecular mass of the enzyme forms were estimated using the elution position of standard molecular mass markers as a reference (not shown). 10.7 mg of fusion protein were applied to the column. The chromatography was performed on a HiLoad Superdex 200 HR column (1.6 cm × 60 cm) from Amersham Biosciences (GE Healthcare, Oslo, Norway), equilibrated and eluted with 20 mm Na‐Hepes, 0.2 m NaCl, pH 7.0 at a flow rate of 0.38 mL·min^−1^ at 4 °C and detection was at 280 nm. The inset represents a SDS/PAGE analysis demonstrating the purity of the fusion proteins after two steps of purification. Lane M, low molecular mass standard (106.5, 97.6, 50.2, 36.9 and 28.9 kDa); lane 2, dimeric form (peak 2); lane 3, monomeric form (peak 3) and lane 4, degradation products (peak 4) after the size‐exclusion chromatography.

The wt and G46S mutant forms of hPAH‐RD were overexpressed in *Escherichia coli* as fusion proteins (MBP‐(pep)_Xa_‐hPAH‐RD) 18. The bacteria were grown at 37 °C and the induction by 1 mm isopropyl‐thio‐β‐d‐galactoside was performed for 8 h at 28 °C. The fusion proteins were purified by affinity chromatography (amylose resin) and centrifuged in a TL‐100 Ultracentrifuge (Beckman, Indianapolis, IN, USA) for 20 min at 50 000 ***g*** before size‐exclusion chromatography (SEC), as described earlier 18. SEC was performed as described in the legend to Fig. 2. The dimeric and monomeric protein fractions were concentrated by Centriplus 30 filter (Amicon, Darmstadt, Germany). The concentration of purified fusion proteins was measured using the absorption coefficient *A*_280_ (1 mg·mL^−1^·cm^−1^) = 1.34. A colorimetric method 19 was in some cases also used to measure enzyme concentrations, with bovine serum albumin as the standard.

### Cleavage of MBP‐hPAH‐RD fusion proteins and assay of self‐association by light scattering

Before cleavage of the MBP‐hPAH‐RD fusion proteins by factor Xa they were centrifuged at 210 000 ***g*** for 15 min at 4 °C. In the standard assay at 20 mm Na‐Hepes, 0.1 m NaCl, pH 7.0 and 25 °C the concentration of the fusion protein was 0.74 mg·mL^−1^, and the concentration of factor Xa was adjusted to give a final ratio (by weight) of 1 : 150 relative to the fusion protein. Self‐association (aggregation) of the factor Xa‐released RD was followed in real‐time by light scattering, as measured by the increase in the apparent absorbance at 350 nm $A_{350}^{\prime }$ = log [*I*_o_/(*I*_p_ + f*I*_d_)], using an Agilent 8453 Diode Array Spectrophotometer (Matriks AS, Oslo, Norway) with a Peltier temperature control unit as previously described 20. The change in light scattering was expressed as Δ$A_{350}^{\prime }$ by subtracting the background absorbance in the absence of the added factor Xa. The rate of oligomerization was expressed as Δ$A_{350}^{\prime }$/Δ*t* and was obtained from the slope of the linear growth phase of each light scattering curve. In each experiment a parallel time‐course cleavage analysis was conducted to rule out any effect of the cleavage rate.

In order to study the inhibitory effect of l‐Phe/d‐Phe on aggregation of MBP‐free wt‐hPAH‐RD and its mutant form, the fusion proteins were preincubated for 5 min at standard assay conditions with l‐Phe (0–1 mm) before the cleavage was initiated with factor Xa. The inhibition of aggregation was analysed by the SigmaPlot^®^ Technical Graphing Software (Alfasoft AS, Lillestrøm, Norway). The Hill plot analyses for the inhibition was performed as previously described 21, and the Hill coefficient (*n*_H_) was calculated by fitting the data into the linear form of the Hill plot equation: log[*v*_i_/(*v*_o_−*v*_i_)] = *n*_H_log[*L*] − *n*_H_log[*k*]. [*L*]_0.5_ represents the concentration of l‐Phe at 50% inhibition.

### SDS/PAGE analyses

The purification of the fusion proteins and the efficiency of its cleavage by factor Xa, was analysed by SDS/PAGE in a 10% (w/v) polyacrylamide gel 22. The gels were stained by Coomassie Brilliant Blue R‐250, scanned using VersaDoc 4000 (Bio‐Rad, Hercules, CA, USA) and quantification of the protein bands was carried out by using the Quantity One 1‐D Analysis Software (Bio‐Rad).

### ANS‐binding assay

Fluorescence‐based ANS binding studies were performed as described 23. The fluorescence emission spectra were recorded between 400 and 600 nm (6‐nm slit width) at 25 °C using an excitation wavelength of 385 nm (6‐nm slit width) on a Perkin‐Elmer LS‐50B luminescence spectrometer (Perkin‐Elmer, Waltham, MA, USA) and by averaging four scans.

### Negative staining of wt‐hPAH‐RD oligomers and electron microscopy

For negative staining EM of wt‐hPAH‐RD oligomers, Formvar‐coated 200 mesh nickel grids (Electron Microscopy Sciences, Hatfield, PA, USA) were used. The grids were further coated with carbon, stored dust‐free in Petri dishes kept at low humidity and glow‐discharged for 15 s prior to use. Negative staining was carried out by first applying 5 μL of a protein solution on the specimen grid. Following absorption for 60 s, the sample drop was removed by blotting with filter paper, and the grid was stained twice with 2% (w/v) aqueous uranyl acetate. After application, the first drop of stain (10 μL) was blotted off immediately, whereafter a fresh drop of the stain was added to the grid for 15 s. After final blotting and drying, the specimens were observed in a Jeol 1230 Electron Microscope (Jeol USA, Inc., Peabody, MA, USA) operated at 80 kV.

## Results

### Overexpression and isolation of the wt‐MBP‐hPAH‐RD fusion proteins

On overexpression of wt‐MBP‐(pep)_Xa_‐hPAH‐RD^1–120^ the soluble affinity purified fusion protein was separated by SEC into oligomeric forms and some aggregates. The chromatogram of the fusion protein (10.7 mg; Fig. 2) revealed four peaks, where peak 1 represented minor aggregates eluted at or near the void volume, while peak 2 and peak 3 represented the dimeric and monomeric forms respectively. Identical mobilities were observed for peaks 2 and 3 on SDS/PAGE (Fig. 2), with an apparent molecular mass of ~ 63 kDa. Peak 4 represents degradation products.

### Cleavage of the MBP stabilized wt fusion proteins

At the standard assay conditions (pH 7.0, 0.1 m NaCl and 25 °C) the cleavage of the metastable and soluble wt‐MBP‐hPAH‐RD fusion proteins (~ 0.7 mg·mL^−1^) by factor Xa (5.0 μg·mL^−1^) was very similar for the dimeric and the monomeric fusion protein, with *t*_1/2_ (time at 50% cleavage) of ~ 11 min. The presence of l‐Phe had no significant effect on the cleavage of wt‐MBP‐hPAH‐RD by factor Xa.

### Aggregation of wt‐hPAH‐RD upon cleavage of dimeric and monomeric MBP fusion proteins and its stereospecific inhibition by l‐Phe

**Figure 3:**
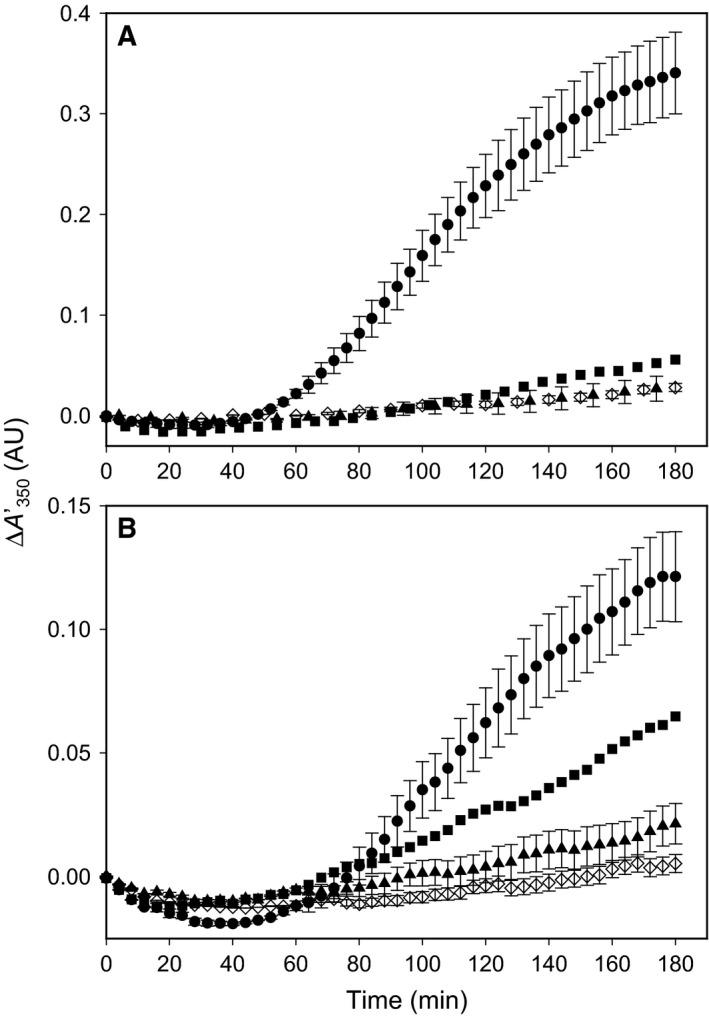
The self‐association of hPAH‐RD ^1–120^ following cleavage of dimeric (A) and monomeric (B) MBP fusion proteins by factor Xa, and the effect of l‐Phe. (A) The time‐course of the self‐association was followed in real‐time by light scattering, as measured by the increase in the apparent absorbance at 350 nm (Δ$A_{350}^{\prime }$). The data points correspond to dimeric wt‐hPAH ^1–120^ fusion protein following cleavage by factor Xa in the absence of any added compound (●) and in the presence of 1 mm l‐Phe (▲). (♢) represents dimeric wt‐hPAH ^1–120^ fusion protein in the absence of factor Xa. (B) The time‐course of the self‐association in the absence of any added compound (●) and in the presence of 1 mm l‐Phe (▲). (♢) represents monomeric wt‐hPAH ^1–120^ fusion protein in the absence of factor Xa. The reactions were performed at standard assay conditions (0.74 mg·mL^−1^ fusion protein, 5.0 μg·mL^−1^ factor Xa, 20 mm Na‐Hepes, 0.1 m NaCl, pH 7.0 and 25 °C). Some data points were omitted for clarity. Error bars represent mean ± SD (*n* = 3).

On cleavage of equal amounts (~ 0.7 mg·mL^−1^) the dimeric and monomeric MBP‐hPAH‐RD fusion proteins by factor X_a_ (5.0 μg·mL^−1^) aggregates are formed (Fig. 3), with a similar time‐course observed for the two fractions. It includes a delay period (lag phase), and a growth phase of increasing light scattering (Δ$A_{350}^{\prime }$/Δ*t*)_max_ of 3.9 ± 0.1 × 10^−3^ and 1.3 ± 0.1 × 10^−3^ AU·min^−1^, for the dimeric (Fig. 3A) and monomeric (Fig. 3B) protein fractions respectively. For ultrastructure of the aggregates and soluble protein (see Fig. 7 below). In the absence of added factor Xa no change in light scattering was observed for the two fractions within the time frame of 3 h.

**Figure 4:**
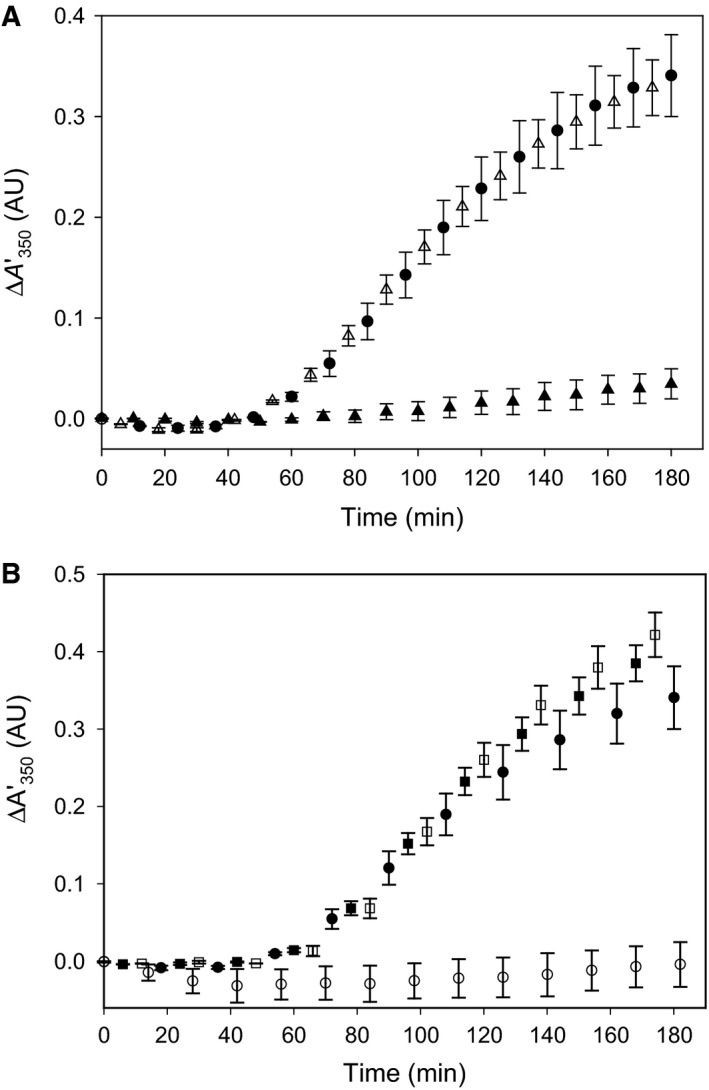
(A) The stereospecific inhibition of the self‐association of hPAH‐RD ^1–120^ by phenylalanine. The time‐course of the self‐association of hPAH‐RD ^1–120^ protein following cleavage of themdimeric fusion protein by factor Xa in the absence (●) and presence of 100 μm l‐Phe (▲) or 100 μm d‐Phe (▵). (B) The self‐association of dimeric hPAH‐RD ^1–120^ and its G46S mutant form and the effect of l‐Phe. The time‐course of the self‐association of hPAH‐RD ^1–120^ dimeric protein following cleavage of the fusion protein by factor Xa in the absence (●) and presence of 150 μm l‐Phe (○); hPAH‐G46S‐RD ^1–120^ dimer fusion protein following cleavage by factor Xa in the absence (■) and presence of 150 μm l‐Phe (□). Some data points were omitted for clarity. The assays were performed at standard assay conditions and error bars represent mean ± SD,* n* = 3 independent experiments.

**Figure 5:**
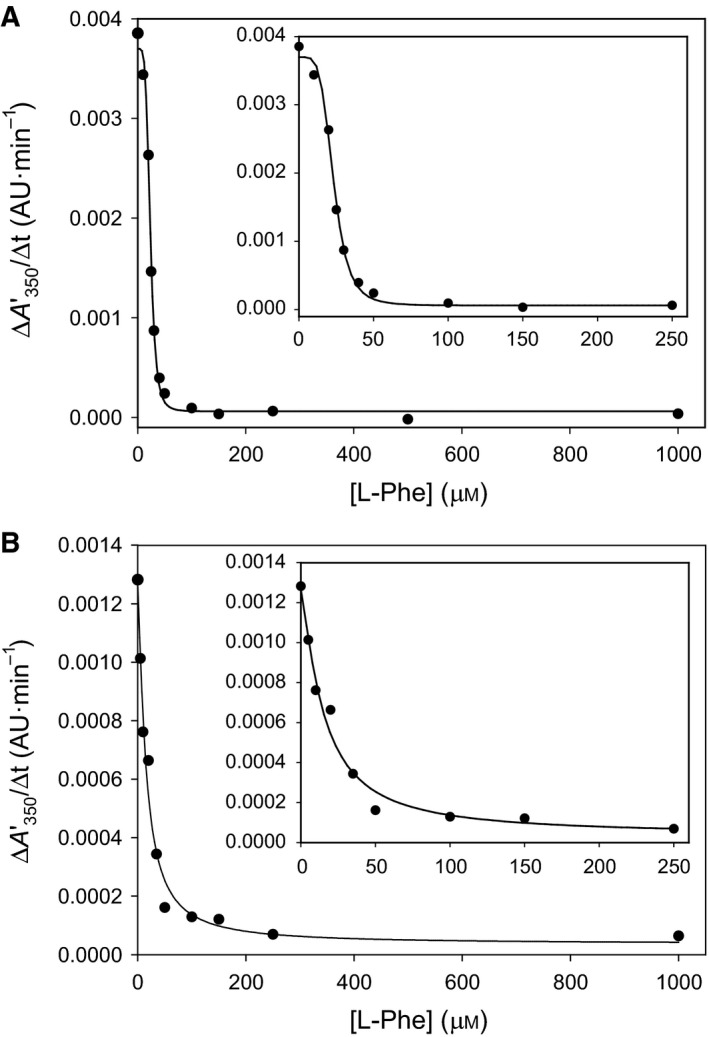
The effect of l‐Phe concentration on the inhibition of the self‐association (aggregation) of dimeric (A) and monomeric (B) MBP fusion protein hPAH‐RD ^1–120^. The inhibitory effect of l‐Phe on the aggregation was assayed at standard conditions (0.74 mg·mL^−1^ fusion protein, 5.0 μg·mL^−1^ factor Xa, 20 mm Na‐Hepes, 0.1 m NaCl, pH 7.0 and 25 °C) with varying concentrations of l‐Phe (0−1 mm) and the rate of forming higher order oligomers (Δ$A_{350}^{\prime }$/Δ*t*) was obtained from the slope of the linear growth phase of each light scattering curve. The l‐Phe dose‐dependent inhibition curves of the aggregation were generated by nonlinear regression analysis of the experimental data using the four parameter logistic equation (see Materials and methods). The insets represent the data obtained at the concentration range 0−250 μm l‐Phe.

The aggregation of the MBP‐free RD is inhibited in a stereospecific manner by l‐Phe; 100 μm l‐Phe almost completely protects against the aggregation, whereas 100 μm d‐Phe and other L‐amino acids gave no effect (Fig. 4A). The inhibition revealed a [*L*]_0.5_ value of 23.3 ± 0.5 μm l‐Phe on cleavage of the dimeric form, with a positive cooperativity; the Hill coefficient was calculated to be about 2 (*n*_H_ = 2.05) (see Materials and methods section). A similar stereospecificity was observed on factor Xa cleavage of the monomeric fraction (data not shown), and the [*L*]_0.5_ value for the inhibition of aggregation was 15.1 ± 2.4 μm l‐Phe, but with a hyperbolic inhibition curve (Fig. 5B).

### Binding of ANS to wt‐MBP‐hPAH‐RD before and after cleavage

**Figure 6:**
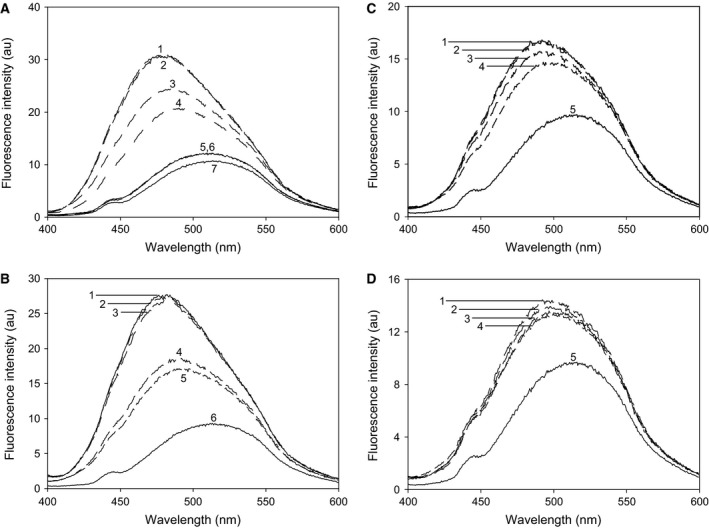
The binding of ANS to dimeric fusion protein MBP‐hPAH‐RD ^1–120^. Briefly, 1.3 μm MBP‐hPAH‐RD was incubated with 60 μm ANS in 20 mm Na‐Hepes, 0.1 m NaCl, pH 7.0 at room temperature for 5 min in the dark. (A) ANS fluorescence emission spectra of the fusion protein in the absence of ligand (trace 1), with 100 μm l‐Phe (*trace* 3), 1 mm l‐Phe (trace 4), and 1 mm d‐Phe (*trace* 2). The MBP protein was used as a control in the absence (trace 5) and in the presence of 1 mm l‐Phe (trace 6) and the emission spectrum of buffer with ANS is shown (trace 7). (B) ANS fluorescence emission spectra observed after cleavage of the dimeric MBP‐hPAH‐RD ^1–120^ fusion protein by factor Xa (*t* = 3 h) in the absence of ligand (trace 1), with 100 μm l‐Phe (trace 4), 1 mm l‐Phe (trace 5), 100 μm d‐Phe (trace 2), 1 mm d‐Phe (trace 3) and the emission spectrum of buffer with ANS (trace 6). (C) ANS fluorescence emission spectra of the monomeric wt‐hPAH ^1–120^ fusion protein in the absence of ligand (trace 1), with 100 μm l‐Phe (trace 3), 1 mm l‐Phe (trace 4), 1 mm d‐Phe (trace 2) and the emission spectrum of buffer with ANS (trace 5). (D) ANS fluorescence emission spectra observed after cleavage of the monomeric wt‐hPAH ^1–120^ fusion protein by factor Xa (*t* = 3 h) in the absence of ligand (trace 1), with 100 μm l‐Phe (trace 3), 1 mm l‐Phe (*trace* 4), 1 mm d‐Phe (trace 2) and the emission spectrum of buffer with ANS (trace 5). The excitation wavelength was 385 nm.

8‐Anilino‐1‐naphthalenesulfonic acid is a spectroscopic probe displaying affinity for hydrophobic clusters which are not tightly packed in a fully folded structure, or become exposed in partially unfolded structures 24. ANS binds to ligand‐free dimeric wt‐MBP‐hPAH‐RD fusion protein (Fig. 6A), and its factor Xa cleaved forms (*t* = 3 h) (Fig. 6B), with an increase in the fluorescence intensity and a blue shift (maximum at ~ 478 nm), resulting from the binding. Identical spectra were obtained in the absence and presence of 1 mm d‐Phe, whereas l‐Phe revealed a concentration‐dependent decrease, in a physiological concentration range, and a red shift of the fluorescence spectra (Fig. 6A,B). A much smaller effect of l‐Phe on ANS binding was observed for the monomeric fusion protein fraction (Fig. 6C,D), which is slightly contaminated by the dimeric form, due to its chromatograpgic tailing. The MBP fusion partner alone has a negligible contribution to the ANS fluorescence, and there was no additional effect of l‐Phe (Fig. 6A).

### l‐Phe does not protect against aggregation of the hPAH‐G46S‐RD mutant form

When the mutant protein hPAH‐G46S‐RD is overexpressed in *E. coli*, mainly insoluble protein is recovered 17. In contrast, here the fusion protein MBP‐hPAH‐G46S‐RD was isolated as a metastable and soluble protein (Fig. 4B). On SEC chromatography only the dimeric form was recovered at sufficiently high yield for further analyses (Fig. 4B), and it revealed the same electrophoretic mobility on SDS/PAGE as the wt dimeric RD. In the absence of added factor Xa no change in light scattering was observed within 3 h. Upon cleavage by factor Xa, the MBP‐free G46S‐RD aggregated, but in this case no protection against aggregation by l‐Phe was observed (Fig. 4B) at concentrations up to 1 mm that inhibited wt‐RD dimer aggregation by > 95% (Fig. 5A).

### Ultrastructure of wt‐hPAH‐RD oligomers

**Figure 7:**
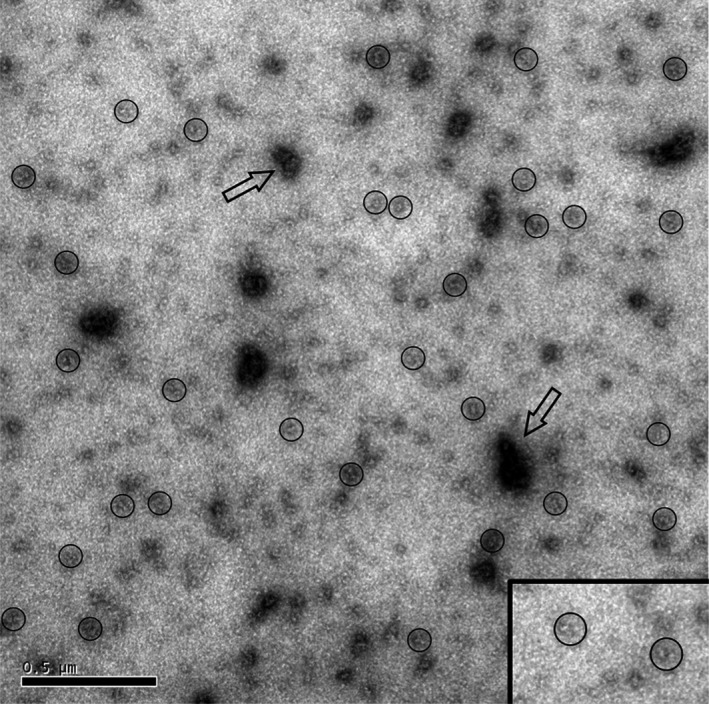
Electron micrograph of MBP‐free wt‐hPAH‐RD ^1–120^ [complete cleavage of its MBP fusion protein by factor Xa (*t* = 3 h)]. The proteins were negatively stained with aqueous uranyl acetate and imaged in the JEOL 1230 Transmission Electron Microscope operated at 80 kV. Structures with a dimeric appearence, corresponding to about 20% of the small‐sized structures are circled and also highlighted in the inset. The open arrows indicate larger protein aggregates. Scale bar: 500 nm.

In order to get information on the fine structure of the wt‐hPAH‐RD^1–120^ protein and its higher order aggregates that are both formed on factor Xa cleavage of the fusion protein, negative staining EM was performed on aliquots removed at different times during the cleavage reaction. EM micrographs corresponding to the final time point (*t* = 180 min) with complete cleavage (Fig. 7) revealed that while the self‐association of the wt protein generated some unstructured higher order aggregates, the main field is dominated by small structures with a dimeric appearance.

## Discussion

A recent SAXS analysis of the rat PAH homotetramer lead to the proposal that the l‐Phe‐induced activation and associated conformational changes involve dimerization of the RDs, creating two binding sites for l‐Phe 8. These binding sites have later been defined in a high resolution (1.8 Å) structure of a homodimeric truncated form of hPAH‐RD in complex with l‐Phe 16. In the same study on size‐exclusion chromatography of hPAH‐RD^1–118^ and hPAH‐RD^19–118^, both constructs are reported to elute as a mixed population of monomer and higher order aggregates. However, the addition of l‐Phe stabilizes the constructs and reduces their aggregation tendency, likely through domain dimerization 16. In the present study, it is found that expressing a similar construct of the human RD as a MBP fusion protein (MBP‐hPAH‐RD) preserves the recombinant protein in a metastable conformational state, which protects against aggregation since MBP functions as a molecular chaperone 25. The dimeric and monomeric fractions, isolated by SEC in an apparent dimer↔monomer equilibrium (Fig. 2), both form aggregates when MBP is cleaved off by factor Xa (Fig. 3). The aggregation of the MBP‐free hPAH‐RDs is prevented by l‐Phe (> 95%) for both forms (Fig. 5), and the effect is stereospecific (Fig. 4a). Half‐maximal inhibition ([*L*]_0.5_) was obtained at 23.3 ± 0.5 μm l‐Phe (cleaved dimeric fraction) and 15.1 ± 2.4 μm l‐Phe (cleaved monomeric fraction). These numbers for the apparent affinity of l‐Phe binding to human RD are in good agreement with the *K*_d_‐value of 15.2 ± 1.1 μm (*n* = 0.9 ± 0.1 sites per monomer) measured for the rat RD dimer by ITC 8). In both the rat and human dimeric forms, a positive cooperativity of l‐Phe binding was observed, with a calculated Hill coefficient (*n*_H_) value of ~ 2.

That l‐Phe also binds to the MBP‐hPAH‐RD dimer before factor Xa cleavage was shown by its reduction of the ANS fluorescence enhancement observed upon the binding of the hydrophobic fluorescence probe to the fusion protein (Fig. 6A). Also, in this assay system, the measured responses to l‐Phe were stereospecific (Figs 4A and 6A), with l‐Phe being present in a physiological concentration range (Fig. 6). This finding indicates that the dimer formation of the fusion protein is based on an interaction between the RDs of two protomers, capable of binding l‐Phe.

In contrast to wt‐MBP‐hPAH‐RD, a structurally/conformationally variant mutant form (MBP‐hPAH‐G46S‐RD, Fig. 1A) revealed no protection against aggregation by l‐Phe upon cleavage by factor Xa of its dimeric form. The lack of stabilizing effect indicates that the binding site in the RD is sensitive to the conformation of its β_1_α_1_β_2_β_3_α_2_β_4_ sandwich fold. In the crystal structure of the hPAH‐RD 16 l‐Phe binding includes the sequence region E^43^xVxAL in the two protomers. The residue G46 is positioned at the entry of α‐helix 1 (A^47^‐E^57^) in a five residue (L^41^‐G^46^) loop structure (loop 1), linking β‐strand 1 and α‐helix 1. The substitution G46→S is predicted to promote a N‐terminal extension of α‐helix 1 by four residues, or one turn 20.

In conclusion, we report a method for the preparation of highly pure, metastable and soluble dimeric↔monomeric forms of the human N‐terminal RD as a MBP fusion protein. Our data support previous biochemical and biophysical studies on isolated recombinant rat and human RDs, that is: (i) l‐Phe binds to a recombinant RD dimer in a physiological concentration range and stabilizes the structure; and (ii) l‐Phe binds with relatively high affinity and with a positive cooperativity (*n*_H_ about 2). In addition, our data demonstrate that the binding is stereospecific for l‐Phe, and that the RD mutation G46S prevents the binding of l‐Phe and its protection against aggregation of MBP‐free RD. This effect is explained by a structural/conformational change in its wt‐binding site involving the residues E^43^xVxAL in dimeric β_1_α_1_β_2_β_3_α_2_β_4_ ACT domain folds. Overall, our data are compatible with an emerging model of the full‐length PAH in which l‐Phe binding to dimerized RDs may be involved in the complex substrate activation process of this multidomain enzyme. However, in contrast to the catalytic domain 13 there is no crystal structure available for the substrate‐bound form of the homotetramer 8, 15.

## Author contributions

JL and TF designed the study; JL, JS and TF analysed the data and wrote the manuscript. All authors have read and approved the manuscript.
